# Mindful Eating, Orthorexic Tendency, and Mediterranean Diet Adherence Identify Weight-Related Eating Profiles in Young Adults

**DOI:** 10.3390/nu18132183

**Published:** 2026-07-05

**Authors:** Giuseppina Augimeri, Domenica Mazza, Luca Gelsomino, Ines Barone, Cinzia Giordano, Stefania Catalano, Carlo Adornetto, Maria Stefania Sinicropi, Daniela Bonofiglio

**Affiliations:** 1 Department of Pharmacy, Health and Nutritional Sciences, University of Calabria, Arcavacata di Rende, 87036 Cosenza, Italy; 2 Centro Sanitario, University of Calabria, Arcavacata di Rende, 87036 Cosenza, Italy; 3 Clinical Laboratory Unit, “Annunziata” Hospital, 87100 Cosenza, Italy; 4 Department of Mathematics, University of Calabria, Arcavacata di Rende, 87036 Cosenza, Italy

**Keywords:** Mediterranean diet, mindful eating, orthorexia nervosa, lifestyle, BMI, obesity prevention

## Abstract

Background: Eating behaviors and psychological processes related to food consumption are increasingly recognized as key factors of successful adherence to a healthy dietary pattern. In this context, mindful eating and adherence to the Mediterranean Diet have emerged as models promoting beneficial health outcomes, whereas orthorexia nervosa, characterized by an obsessive focus on eating healthy foods, often leads to dietary inadequacies. Here, we investigated the association among mindful eating, adherence to the Mediterranean Diet, orthorexia nervosa and BMI in a cohort of university students. Methods: A sample of 518 Italian university students completed an online survey assessing general and sociodemographic characteristics, the adherence to the Mediterranean Diet pattern by MEDAS and MEDLIFE, the orthorexia tendency by ORTO-15 test and the degree of mindful eating by the Mind-Eat Scale. Students’ *t*-test, chi-square test, Pearson’s correlation, and K-means cluster analysis were used for analyses. Results: The participants had a mean BMI of 23.08 ± 3.51, with 69% normal-weight and 25% overweight/obese individuals, showing statistically significant sex differences. Mean Mind-Eat, MEDAS, MEDLIFE and ORTO-15 scores were 3.22 ± 0.46, 8.81 ± 2.35, 3.06 ± 1.14 and 38.31 ± 5.94, respectively. Men scored significantly higher than women in all categories, except for the MEDAS score, where no sex-related differences were observed. Interestingly, the Mind-Eat score was positively associated with ORTO-15 (r = 0.24, *p* < 0.0001), MEDAS (r = 0.23, *p* < 0.0001), and MEDLIFE (r = 0.11, *p* = 0.01) scores. Three clusters were derived, distinguishing optimal (cluster 2), intermediate (cluster 1) and poor (cluster 3) eating profiles, showing sex differences. BMI was significantly lower in cluster 2 than in cluster 3. Conclusions: Our results suggest that integrating psychological and dietary indicators of eating behavior may help identify young adults with less favorable weight-related profiles.

## 1. Introduction

Adherence to a healthy dietary lifestyle plays a crucial role in the prevention of several physical and psychological health conditions. Traditionally, health outcomes have been primarily related to dietary intake and nutrient composition. Recently, the importance of eating habits and psychological processes related to food consumption has emerged as a crucial factor in promoting a healthy dietary lifestyle [1]. Thus, different “non-dieting” techniques have been developed to improve healthy eating and promote weight management, including mindful eating. Mindful eating applies the principles of mindfulness, including awareness, acceptance and decentering, to dietary habits, based on the concept that a health status is associated not only with what is eaten, but also with how it is eaten [1]. Awareness of thoughts and feelings about food, and responsiveness to hunger and satiety cues, are considered key factors in mindful eating [2,3]. Several studies have reported that mindful eating interventions are associated with weight loss and improved cardiovascular biomarkers [4,5,6]. Moreover, a higher degree of mindful eating is associated with a reduced risk of eating-disorder symptoms, including binge eating and emotional eating [7].

In contrast to mindful eating, orthorexia nervosa, an obsession with consuming foods that are considered healthy or clean, is associated with poor emotional regulation, anxious attachment to food and a negative self-perceived body image [8]. Orthorexia nervosa leads to a very strict diet, causing nutritional deficiencies and social isolation [9]. A negative association has been found between orthorexic tendencies and mindful eating [8,10,11], although the research in this field is still limited.

While mindful eating and orthorexia nervosa describe different psychological relationships with food, they do not directly capture the overall quality of individuals’ dietary patterns. For this reason, integrating psychological dimensions of eating behavior with objective measures of dietary adherence may provide a more comprehensive understanding of health-related eating profiles.

The Mediterranean Diet (MD) has been described as the strongest evidence-based healthy dietary model for weight management. It is based on high consumption of fruits, vegetables and whole grains, moderate intake of dairy products, eggs, fish and white meat and low consumption of red meat and sweets. Most importantly, the MD pattern encourages a healthy lifestyle, including adequate rest, regular physical activity, conviviality and the consumption of seasonal and local foods [12]. High adherence to the MD is associated with a reduced risk of developing various metabolic and chronic-degenerative diseases, including type 2 diabetes, obesity, cardiovascular diseases and cancer [13,14,15,16,17]. Moreover, the MD has been described as having a positive role in various psychological disorders, including depression and anxiety [18,19]. Indeed, fruits and vegetables, which represent the main components of the MD, are rich in antioxidants and polyphenols that reduce oxidative stress and inflammation implicated in the onset of mental disorders [20]. Moreover, fiber and proteins from MD foods influence the composition of the gut microbiota, improving the progression of mental diseases through the regulation of the gut–brain axis [21].

Although previous studies have investigated mindful eating behaviors, orthorexic tendencies, and adherence to the MD independently, limited research has simultaneously explored how these psychological and dietary dimensions interact within the same population and modulate weight management. In particular, identifying modifiable psychological and dietary factors associated with BMI is crucial in young adulthood since this life stage influences long-term eating behaviors and weight-related trajectories. Mindful eating represents an adaptive psychological approach to food, whereas orthorexic tendency reflects a potentially maladaptive pattern in which the pursuit of healthy eating may become rigid, perfectionistic, and emotionally distressing. Mediterranean Diet adherence, on the other hand, captures the overall quality of the dietary pattern and lifestyle habits. Therefore, integrating these psychological and dietary dimensions may help distinguish individuals who share similar dietary scores but differ in their psychological relationship with food, or vice versa. Indeed, we hypothesized that higher mindful eating would be associated with greater adherence to the Mediterranean Diet, lower orthorexic tendency, and more favorable BMI values. Thus, the aim of this cross-sectional study was to investigate the relationship among mindful eating, orthorexic tendency, adherence to the MD pattern and BMI in a cohort of university students and to identify distinct eating profiles based on the integration of psychological and dietary approaches.

## 2. Materials and Methods

### 2.1. Participants

**Figure 1:**
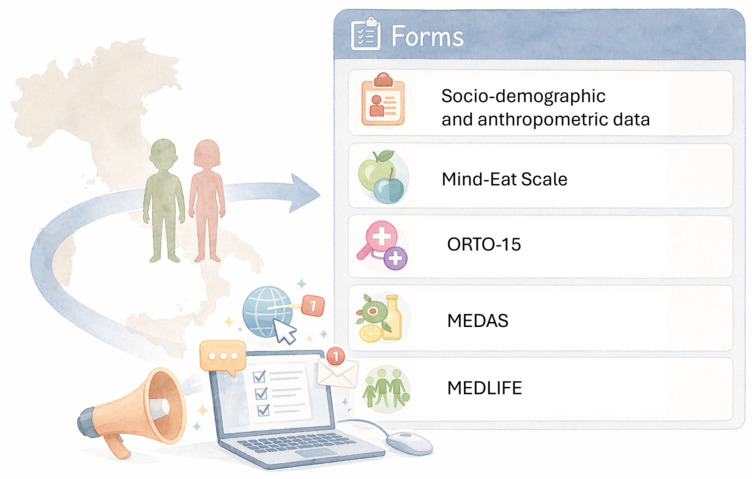
Schematic overview of the study design.

A total of 518 participants, including 156 men and 362 women, were recruited via social media platforms (Facebook 568.0.0, Instagram 437.0.0) and instant messaging apps, such as WhatsApp 26.22.76, for this cross-sectional study. Because the survey link was disseminated through open online channels, a formal response rate could not be calculated. Participants were informed of the study’s purpose and hypotheses and invited to complete a web survey hosted on the Google platform, after giving informed consent. The inclusion criteria required participants to be healthy individuals over the age of 18, recruited from Italian university communities, who provided informed consent and completed the online questionnaire. Students attended several faculties, including health sciences, engineering and technological sciences, economic and legal sciences, natural sciences, social and political sciences and the humanities. Responses were not included in the final analyses when participants were younger than 18 years, did not provide informed consent or did not belong to the target university population. This study was approved by the Italian Ethics Committee of the University of Calabria, Italy (#2025-UCALPRG-0147665-20/06/2025). The schematic overview of the study design is reported in Figure 1.

### 2.2. Demographic and Anthropometric Information

The first part of the questionnaire focused on the general characteristics of the participants. Participants were asked to provide demographic information, including age, sex and university department affiliation, as well as height and weight to calculate BMI.

### 2.3. Scales

#### 2.3.1. Mind-Eat Scale

The Mind-Eat Scale is a self-report questionnaire composed of 24 items, assessing levels of mindful eating and its subdimensions in the general population. The questionnaire is composed of six dimensions covering the key features of the mindful eating approach, including non-reactivity, awareness, openness, gratitude, non-judgment, and hunger/satiety. Every dimension is explored by 4 items rated on a 5-point frequency scale. Negatively worded items, including 2, 4, 5, 8, 10, 17, 20, and 22 items, were reverse-coded prior to analysis. The Mind-Eat score was calculated as the average of the six subscale scores. A higher Mind-Eat score indicated a higher level of mindful eating [22].

#### 2.3.2. ORTO-15

The ORTO-15 questionnaire is a validated tool used to assess orthorexic tendencies, based on 15 questions with Likert-scale response categories (always, often, sometimes, or never) scored from 1 to 4 [23]. According to the original scoring procedure, lower ORTO-15 raw scores indicate greater orthorexic tendency, whereas higher scores indicate lower orthorexic tendency. Therefore, in correlation and regression analyses, the original ORTO-15 total score was retained and interpreted accordingly. For the cluster analysis, a reversed ORTO-15 score was computed to align the directionality of the variables, so that higher reversed ORTO-15 z-scores indicated greater orthorexic tendency.

#### 2.3.3. Mediterranean Diet Adherence Screener (MEDAS) and Mediterranean Lifestyle (MEDLIFE) Index

Adherence to the MD pattern was evaluated by administering the Mediterranean Diet Adherence Scale Screener (MEDAS) [24] and the Mediterranean Lifestyle (MEDLIFE) [25] questionnaires. MEDAS comprises 14 items, including 2 questions on food consumption habits and 11 questions on food consumption frequency. The MEDAS score ranged from 0 to 14, as each question is scored on a scale of 0 to 1. MEDLIFE is composed of 6 questions investigating lifestyle habits such as engagement in physical activity, sleep, and social and conviviality habits. Each question was scored 0 or 1, resulting in a total score ranging from 0 to 6.

### 2.4. Statistical Analysis

Data were exported from the Google platform and analyzed using IBM SPSS Statistics version 25.0 for Windows. Data were reported as the mean and SD and statistical differences between samples were evaluated using Student’s *t*-test for continuous variables and the chi-square test for categorical variables. The association between the Mind-Eat score and the collected variables was assessed using univariate, multivariable linear regression models, and Pearson’s and partial correlations in IBM SPSS Statistics version 25.0. K-means cluster analysis was conducted using standardized variables (z-scores) of the MEDAS, Mind-Eat and ORTO-15 scores. To assess the robustness of the clustering solution, a repeated cross-validation procedure was performed over 100 iterations. At each iteration, the clustering algorithm was re-applied to a resampled subset of the data, and both clustering quality and stability were evaluated using the Silhouette Score and the Adjusted Rand Index (ARI). The Silhouette Score ranges from −1 to 1, where values close to 1 indicate well-separated and cohesive clusters, values around 0 indicate overlapping clusters, and negative values suggest that observations may have been assigned to the wrong cluster. The Adjusted Rand Index ranges from −1 to 1, with 1 indicating perfect agreement between two clustering solutions, 0 corresponding to agreement expected by chance, and negative values indicating less agreement than would be expected by chance. Therefore, while the Silhouette Score assesses clustering quality, the ARI evaluates the stability and reproducibility of the clustering structure across repeated runs.

The cross-validation analysis yielded a mean Silhouette Score of 0.260 (variance = 2.35 × 10^−5^) and a mean ARI of 0.887 (variance = 4.54 × 10^−3^). Although the Silhouette Score indicates a moderate level of cluster separation, its extremely low variance across iterations demonstrates that the clustering quality remains highly consistent under data resampling. Furthermore, the high mean ARI value (≈0.89), coupled with its low variance, indicates a strong agreement among clustering solutions obtained in different cross-validation runs. Overall, these results provide evidence that the identified clustering structure is robust and stable, with limited sensitivity to sampling variability and a high degree of reproducibility. Differences in continuous variables across clusters were assessed using the Kruskal–Wallis test. When a significant overall effect was detected, pairwise comparisons were performed using the Mann–Whitney U test with Bonferroni correction or the chi-square test. *p*-values lower than 0.05 were considered statistically significant. Analyses were conducted using IBM SPSS Statistics version 25.0 and GraphPad Prism 7.

## 3. Results

### 3.1. General Characteristics in the Study Population

As shown in Table 1, the study population consisted of 518 participants, including 156 men and 362 women, with a mean age of 23 ± 2.86 years and no significant sex differences. Mean BMI falls within the normal range, with men having a higher BMI than women (*p* = 0.001). Most students were classified as normal weight (69%), followed by overweight (20%), underweight (6%) and obese (5%). When the population was categorized by sex and BMI, sex-related differences were found, showing a higher percentage of overweight and obesity in men than women, whereas underweight was more frequent in women than men (*p* < 0.0001). Participants were mainly enrolled in health science faculties (42%), followed by engineering and technological sciences (14%), natural sciences (11%), economic and legal sciences (9%), the humanities (9%), social and political sciences (8%), and other faculties (8%). Sex-related differences in the faculty distribution were observed with a higher proportion of women attending health science faculties and a greater proportion of men attending engineering and technological sciences faculties (*p* < 0.0001).

### 3.2. Mind-Eat Score Across the Six Dimensions of Mindful Eating in the Total Sample Population and Categorized by Sex

The mean Mind-Eat score was 3.22 ± 0.46 in the total population, with men having a significantly higher score than women (3.29 ± 0.46 vs. 3.19 ± 0.46, *p* = 0.02), suggesting a higher level of mindful eating. Analysis of the mean scores across the six dimensions of mindful eating showed that sex significantly influenced non-reactivity (*p* = 0.009), openness (*p* = 0.005) and non-judgment (*p* < 0.0001) (Table 2).

### 3.3. Association Among Mind-Eat, ORTO-15, MEDAS and MEDLIFE Scores in the Study Cohort

MEDAS and MEDLIFE scores, which assess adherence to the MD pattern, were 8.81 ± 2.35 and 3.06 ± 1.14, respectively, in the total population, indicating medium adherence to the MD. When categorizing the population by sex, men showed a significantly higher MEDLIFE score than women (*p* = 0.004), whereas no sex-related differences were found in the MEDAS score. The ORTO-15 score was 38.31 ± 5.94, with men having a higher score than women (*p* = 0.02) (Table 3).

Partial correlation analyses controlling for sex, BMI and faculty showed a positive association between the Mind-Eat score and MD pattern scores (MEDAS: r = 0.23, *p* < 0.001; MEDLIFE: r = 0.11, *p* < 0.001). Mindful eating was also positively associated with ORTO-15 score (r = 0.24, *p* < 0.0001), indicating lower orthorexic tendency among individuals with higher mindful eating levels (Table 4).

In multivariable regression analysis, using the Mind-Eat score as the dependent variable and the MEDAS, MEDLIFE and ORTO-15 scores, along with sex and BMI as independent variables, mindful eating was positively associated with ORTO-15 (β = 0.23, *p* < 0.0001) and MEDAS (β = 0.25, *p* < 0.0001) scores, negatively associated with BMI (β = −0.17, *p* < 0.0001) and lower in women (sex = W, β = −0.11, *p* = 0.01) (Table 5).

### 3.4. Cluster-Derived Eating Behavior Profiles in the Study Population

**Figure 2:**
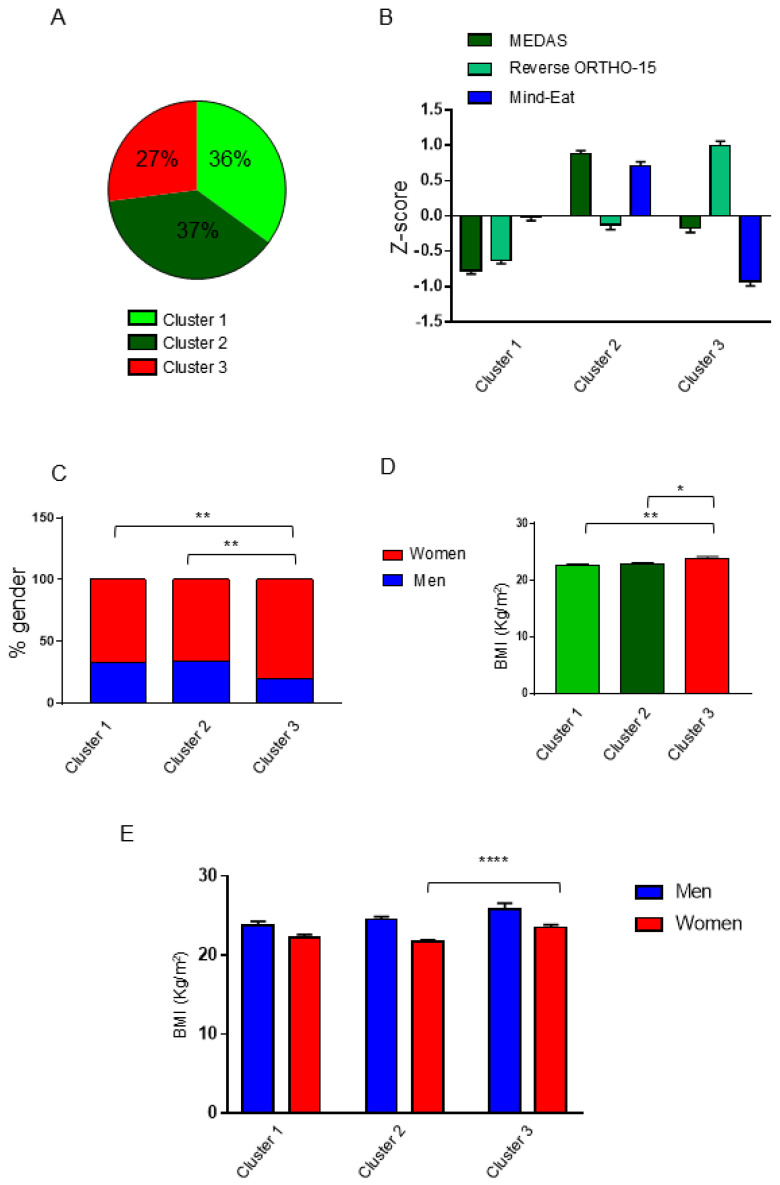
(**A**) The distribution of data-driven clusters in study cohort. (**B**) MEDAS, Reverse-ORTO-15 and Mind-Eat z-scores among the three clusters. (**C**) Sex distribution for each cluster in the study population. (**D**) Mean BMI for each cluster in the study population. (**E**) Mean BMI for each cluster in the study population, categorized by sex. * *p* < 0.05, ** *p* < 0.005, **** *p* < 0.0001.

Clustering analysis, using the standardized MEDAS, Reverse ORTO-15 and Mind-Eat z-scores as variables, revealed the presence of three clusters composed of 36% (*n* = 181), 37% (*n* = 197) and 27% (*n* = 140) of participants (Figure 2A). Cluster 1 was characterized by participants with the lowest z-scores, indicating an intermediate eating profile; cluster 2 included subjects with high MEDAS and Mind-Eat z-scores and a low reversed ORTO-15 z-score, representing the optimal eating profile; cluster 3 included participants with low MEDAS and Mind-Eat z-scores and a high reversed ORTO-15 z-score, suggesting a poor eating profile (Figure 2B). A significantly higher percentage of women was found in cluster 3 compared to clusters 1 and 2 (Figure 2C). Moreover, BMI was significantly higher in cluster 3 (23.88 ± 3.91) than in clusters 1 (22.66 ± 3.33) and 2 (22.89 ± 3.27) (Figure 2D). When categorizing the population by sex, we found a significantly higher BMI in women included in cluster 3 (23.51 ± 3.86) than in those included in cluster 2 (21.69 ± 2.78) (Figure 2E).

## 4. Discussion

In this study, we identified three distinct eating behavior profiles, namely, poor, intermediate and optimal eating profiles in a cohort of university students based on their adherence to the MD, mindful eating and orthorexic tendency.

Our study population had a medium level of mindful eating, with men showing a higher score than women. In particular, men had higher mean scores in the dimension related to non-reactivity, openness and non-judgment. Our data are in line with the literature, which reports that men have a more open approach to food experiences and less emotional reactivity. In contrast, women, who are more vulnerable to socio-cultural beauty standards [26], tend to respond to emotional stress by eating [22].

When adjusting for sex and BMI, the Mind-Eat score was positively associated with adherence to the MD and negatively associated with the orthorexic tendency. To the best of our knowledge, this is the first study to simultaneously examine the relationship among these psychological and dietary dimensions.

Mindful eating and orthorexia have some common aspects, but the line between healthy and pathological approaches toward food is often difficult to define. Indeed, eating awareness is a feature of healthy eating behavior. However, when it becomes a rigid or perfectionistic eating attitude, such as in orthorexia nervosa, it turns into a maladaptive preoccupation leading to guilt, anxiety, and self-imposed rules.

To date, only one study has investigated the relationship between mindful eating and adherence to the MD, demonstrating a positive correlation between eating behavior and the dietary pattern in Turkish young adults [27].

In our study population, only the MEDAS, but not the MEDLIFE score, was significantly associated with mindful eating in the multivariable regression model, suggesting a more specific link between eating and dietary habits rather than with the broader lifestyle MD pattern.

Conflicting data have been reported on the relationship between mindful eating and orthorexia nervosa. Most studies have demonstrated a negative association between mindful eating and orthorexia nervosa, which is mediated by psychological factors such as perfectionism, guilt, shame, emotion dysregulation and self-compassion [8,11,28,29]. Other authors have found a positive association between orthorexia nervosa and mindful eating. For instance, Dobos et al. found that mindful eating is a predictor of social media addiction, orthorexia nervosa and emotion dysregulation [30].

ORTO-15 was used in the present study because it is one of the earliest and most widely applied questionnaires for assessing orthorexic eating tendencies, thereby allowing comparison with previous studies in non-clinical and university populations. Importantly, ORTO-15 was used only as an indicator of orthorexic tendency rather than to diagnose orthorexia nervosa. Indeed, the psychometric properties and diagnostic validity of ORTO-15 have been debated, and the scale may not adequately distinguish between a non-pathological interest in healthy eating and clinically relevant orthorexic pathology. Future studies should replicate these findings using more recent instruments, such as the Düsseldorf Orthorexia Scale [31] and the Teruel Orthorexia Scale [32].

Interestingly, using the standardized scores of MEDAS, Mind-Eat and ORTO-15, three different eating profiles were found in a clustering analysis. The poor eating profile was characterized by low adherence to the MD and low mindful eating, along with a high tendency toward orthorexia nervosa; the optimal eating profile showed high adherence to the MD and mindful eating with low orthorexic tendency, whereas the intermediate profile had features positioned between these two extremes. We found a higher percentage of women in the poor eating profile, which is consistent with the data from other authors reporting that women have a higher level of emotional eating, particularly in response to stress [33]. Of note, we observed a significantly higher BMI in the poor eating profile than in the other profiles. These data are in line with our previous findings on the association between orthorexia nervosa and anthropometric factors, showing that this eating behavior is positively associated with female sex and negatively associated with BMI [34]. Moreover, the association between high adherence to the MD and BMI values has been widely investigated [13,35,36,37,38,39,40,41]. In our previous findings, we demonstrated that adherence to the MD is positively associated with the skin carotenoid score measured by the Veggie Meter^®^. Interestingly, skin carotenoids were negatively associated with BMI across different age groups [42,43,44,45,46], suggesting that higher carotenoid status, reflecting higher adherence to an MD pattern rich in fruit and vegetable consumption, is associated with a more favorable weight status. Moreover, recently, the 20-year follow-up of the ATTICA cohort study conducted on 1582 participants revealed that MD adherence and its changes are inversely related to BMI at 20 years. Interestingly, maintaining a high adherence to the MD was associated with a reduced risk of being overweight or obese [47].

From a practical perspective, the identification of distinct eating behavior profiles may help inform health promotion strategies in university settings. Students characterized by lower MD adherence, lower mindful eating, and greater orthorexic tendency may benefit from integrated preventive interventions addressing both dietary quality and the psychological dimensions of eating behavior. Rather than focusing exclusively on nutritional information, university-based programs could include mindful eating strategies, education on flexible adherence to healthy dietary patterns, and support for reducing rigid food rules and weight-related concerns. However, these profiles should be interpreted as exploratory and not as diagnostic categories.

Several study limitations should be acknowledged. First, the cross-sectional design prevents causal or directional inferences among mindful eating, orthorexic tendency, MD adherence, and BMI. Thus, it cannot be determined whether eating behavior profiles influence BMI, whether BMI affects psychological and dietary attitudes toward food, or whether these associations are partly explained by unmeasured confounding factors. Second, anthropometric parameters were self-reported, which may have introduced reporting bias in BMI estimation. Third, participants were recruited through social media platforms and instant messaging applications using a convenience sampling approach. As a result, the response rate could not be calculated, and the sample may not be representative of the broader population of Italian university students. Fourth, the predominance of female participants and the relatively high proportion of students from health-related faculties may have influenced the observed eating behavior profiles, affecting generalizability to broader or more diverse populations.

## 5. Conclusions

Taken together, our data highlight the multidimensional nature of eating behavior. Importantly, the unfavorable profile was associated with higher BMI, suggesting that the integration of psychological and dietary indicators may help identify individuals with poor weight-related characteristics. Further longitudinal studies are needed to investigate whether a holistic approach to eating behavior might impact long-term obesity prevention.

## Figures and Tables

**Table 1:** General characteristics of the study population.

	Total *n* = 518	Men *n* = 156	Women *n* = 362	*p*-Value
Age (years, mean ± SD)	23 ± 2.86	22.99 ± 2.86	23 ± 2.86	0.97
Weight (kg, mean ± SD)	64.90 ± 13.41	77.01 ± 12.65	59.68 ± 9.91	<0.0001
Height (cm, mean ± SD)	166.97 ± 10.02	177.14 ± 6.81	162.59 ± 7.73	<0.0001
BMI (kg/m^2^, mean ± SD)	23.08 ± 3.51	24.49 ± 3.45	22.48 ± 3.36	<0.0001
Underweight (*n* %)	29, 6	4, 2	25, 7	<0.0001
Normal weight (*n*, %)	360, 69	87, 56	274, 75	
Overweight (*n*, %)	103, 20	53, 34	50, 14	
Obese (*n*, %)	26, 5	12, 8	13, 4	
Faculty (*n*, %)				
Health Sciences	217, 42	53, 34	164, 45	
Engineering and Technological Sciences	72, 14	39, 24	33, 9	
Economic and Legal Sciences	45, 9	15, 10	30, 8	0.0001
Natural Sciences	56, 11	20, 13	36, 10	
Social and Political Sciences	39, 8	4, 3	35, 10	
Humanities	46, 9	11, 7	35, 10	
Other	43, 8	14, 9	29, 8	

BMI: Body Mass Index.

**Table 2:** Mind-Eat score in the total population and categorized by sex.

	Total	Men	Women	*p*-Value
Mind-Eat score	3.22 ± 0.46	3.29 ± 0.46	3.19 ± 0.46	0.02
Awareness	3.43 ± 0.86	3.40 ± 0.90	3.44 ± 0.85	0.68
Non-reactivity	2.94 ± 0.77	3.08 ± 0.85	2.89 ± 0.73	0.009
Openness	3.18 ± 0.78	3.33 ± 0.81	3.12 ± 0.76	0.005
Gratitude	3.38 ± 0.86	3.28 ± 0.89	3.42 ± 0.84	0.08
Non-judgment	2.98 ± 0.80	3.28 ± 0.72	2.85 ± 0.80	<0.0001
Hunger/Satiety	3.40 ± 0.84	3.40 ± 0.85	3.40 ± 0.84	0.96

**Table 3:** MEDAS, MEDLIFE and ORTO-15 scores in our total sample population and categorized by sex.

Score ± SD	Total	Men	Women	*p*-Value
MEDAS	8.81 ± 2.35	9.08 ± 2.42	8.7 ± 2.32	0.09
MEDLIFE	3.06 ± 1.14	3.28 ± 1.21	2.97 ± 1.09	0.004
ORTO-15	38.31 ± 5.94	39.22 ± 5.8	37.93 ± 5.97	0.02

**Table 4:** Partial correlation analysis between Mind-Eat score and MEDAS, MEDLIFE and ORTO-15 scores.

	r	*p*-Value
MEDAS	0.23	<0.0001
MEDLIFE	0.11	0.01
ORTO-15	0.24	<0.0001

**Table 5:** Multivariable regression analysis between Mind-Eat score and several parameters.

Score ± SD	β (CI 95%)	*p*-Value
Sex [W = 1]	−0.11 (−0.42; −0.05)	0.01
BMI	−0.17 (−0.07; −0.03)	<0.0001
ORTO-15	0.23 (0.02; 0.05)	<0.0001
MEDAS	0.25 (0.07; 0.14)	<0.0001
MEDLIFE	0.01 (−0.06; 0.09)	0.72

## Data Availability

All data generated or analyzed during this study are included in this article. Further enquiries can be directed to the corresponding author.

## Abbreviations

The following abbreviations are used in this manuscript:

BMI	Body Mass Index
CI	Confidence Interval
MD	Mediterranean Diet
MEDAS	Mediterranean Diet Adherence Screener/Mediterranean Diet Adherence Scale Screener
MEDLIFE	Mediterranean Lifestyle Index
SD	Standard Deviation
z-score	Standard Score

## References

[1] Tapper K. (2022). Mindful eating: What we know so far. Nutr. Bull..

[2] Mantzios M. (2023). Mindful eating: A conceptual critical review of the literature, measurement and intervention development. Nutr. Health.

[3] Peitz D., Schulze J., Warschburger P. (2021). Getting a deeper understanding of mindfulness in the context of eating behavior: Development and validation of the Mindful Eating Inventory. Appetite.

[4] Daly P., Pace T., Berg J., Menon U., Szalacha L.A. (2016). A mindful eating intervention: A theory-guided randomized anti-obesity feasibility study with adolescent Latino females. Complement. Ther. Med..

[5] Mason A.E., Epel E.S., Kristeller J., Moran P.J., Dallman M., Lustig R.H., Acree M., Bacchetti P., Laraia B.A., Hecht F.M. (2016). Effects of a mindfulness-based intervention on mindful eating, sweets consumption, and fasting glucose levels in obese adults: Data from the SHINE randomized controlled trial. J. Behav. Med..

[6] Daubenmier J., Epel E.S., Moran P.J., Thompson J., Mason A.E., Acree M., Goldman V., Kristeller J., Hecht F.M., Mendes W.B. (2019). A Randomized Controlled Trial of a Mindfulness-Based Weight Loss Intervention on Cardiovascular Reactivity to Social-Evaluative Threat Among Adults with Obesity. Mindfulness.

[7] Sala M., Shankar Ram S., Vanzhula I.A., Levinson C.A. (2020). Mindfulness and eating disorder psychopathology: A meta-analysis. Int. J. Eat. Disord..

[8] Strahler J. (2021). Trait mindfulness differentiates the interest in healthy diet from orthorexia nervosa. Eat. Weight. Disord..

[9] Gkiouleka M., Stavraki C., Sergentanis T.N., Vassilakou T. (2022). Orthorexia Nervosa in Adolescents and Young Adults: A Literature Review. Children.

[10] Kalika E., Hussain M., Egan H., Mantzios M. (2023). Exploring the moderating role of mindfulness, mindful eating, and self-compassion on the relationship between eating-disordered quality of life and orthorexia nervosa. Eat. Weight. Disord..

[11] Miley M., Egan H., Wallis D., Mantzios M. (2022). Orthorexia nervosa, mindful eating, and perfectionism: An exploratory investigation. Eat. Weight. Disord..

[12] Sofi F., Martini D., Angelino D., Cairella G., Campanozzi A., Danesi F., Dinu M., Erba D., Iacoviello L., Pellegrini N. (2025). Mediterranean diet: Why a new pyramid? An updated representation of the traditional Mediterranean diet by the Italian Society of Human Nutrition (SINU). Nutr. Metab. Cardiovasc. Dis..

[13] Rodríguez Núñez S., Rubín-García M., Martín-Sánchez V., Álvarez-Álvarez L., Molina A.J. (2025). Mediterranean Diet, Obesity-Related Metabolic Cardiovascular Disorders, and Environmental Sustainability: A Systematic Review. Nutrients.

[14] Zheng X., Zhang W., Wan X., Lv X., Lin P., Si S., Xue F., Wang A., Cao Y. (2024). The effects of Mediterranean diet on cardiovascular risk factors, glycemic control and weight loss in patients with type 2 diabetes: A meta-analysis. BMC Nutr..

[15] Zupo R., Castellana F., Piscitelli P., Crupi P., Desantis A., Greco E., Severino F.P., Pulimeno M., Guazzini A., Kyriakides T.C. (2023). Scientific evidence supporting the newly developed one-health labeling tool “Med-Index”: An umbrella systematic review on health benefits of Mediterranean diet principles and adherence in a Planeterranean perspective. J. Transl. Med..

[16] Dinu M., Pagliai G., Casini A., Sofi F. (2018). Mediterranean diet and multiple health outcomes: An umbrella review of meta-analyses of observational studies and randomised trials. Eur. J. Clin. Nutr..

[17] Ceraudo F., Caparello G., Galluccio A., Avolio E., Augimeri G., De Rose D., Vivacqua A., Morelli C., Barone I., Catalano S. (2022). Impact of Mediterranean Diet Food Choices and Physical Activity on Serum Metabolic Profile in Healthy Adolescents: Findings from the DIMENU Project. Nutrients.

[18] Jacka F.N., O’Neil A., Opie R., Itsiopoulos C., Cotton S., Mohebbi M., Castle D., Dash S., Mihalopoulos C., Chatterton M.L. (2017). A randomised controlled trial of dietary improvement for adults with major depression (the ‘SMILES’ trial). BMC Med..

[19] Parletta N., Zarnowiecki D., Cho J., Wilson A., Bogomolova S., Villani A., Itsiopoulos C., Niyonsenga T., Blunden S., Meyer B. (2019). A Mediterranean-style dietary intervention supplemented with fish oil improves diet quality and mental health in people with depression: A randomized controlled trial (HELFIMED). Nutr. Neurosci..

[20] Pereira G.A., da Silva A., Hermsdorff H.H.M., Moreira A.P.B., de Aguiar A.S. (2021). Association of dietary total antioxidant capacity with depression, anxiety, and sleep disorders: A systematic review of observational studies. J. Clin. Transl. Res..

[21] Kabthymer R.H., Karimi L., Livesay K., Lee M., Apostolopoulos V., Millar R., McKay S., Barry S., Olga C.N., Colomer M.F. (2025). Effect of Mediterranean diet on mental health outcomes: A systematic review. Nutr. Res. Rev..

[22] Van Beekum M., Shankland R., Rodhain A., Robert M., Marchand C., Herry A., Prioux C., Touvier M., Barday M., Turgon R. (2024). Development and validation of the mindful eating scale (mind-eat scale) in a general population. Appetite.

[23] Donini L.M., Marsili D., Graziani M.P., Imbriale M., Cannella C. (2005). Orthorexia nervosa: Validation of a diagnosis questionnaire. Eat. Weight. Disord..

[24] García-Conesa M.T., Philippou E., Pafilas C., Massaro M., Quarta S., Andrade V., Jorge R., Chervenkov M., Ivanova T., Dimitrova D. (2020). Exploring the Validity of the 14-Item Mediterranean Diet Adherence Screener (MEDAS): A Cross-National Study in Seven European Countries around the Mediterranean Region. Nutrients.

[25] Sotos-Prieto M., Santos-Beneit G., Bodega P., Pocock S., Mattei J., Peñalvo J.L. (2015). Validation of a questionnaire to measure overall Mediterranean Lifestyle habits for research application: The Medliterranean Lifestyle Index (MEDLIFE). Nutr. Hosp..

[26] Moradi B. (2010). Addressing Gender and Cultural Diversity in Body Image: Objectification Theory as a Framework for Integrating Theories and Grounding Research. Sex. Roles.

[27] Ongun Yilmaz H., Arslan S., Tari Selcuk K., Yilmaz S. (2026). Association Between Mediterranean Diet Adherence and Intuitive and Mindful Eating in Turkish Young Adults. Nutrients.

[28] Thorne J., Hussain M., Mantzios M. (2023). Exploring the relationship between orthorexia nervosa, mindful eating and guilt and shame. Health Psychol. Rep..

[29] Barakat M., Salim N.A., Malaeb D., Dabbous M., Sakr F., Hallit S., Fekih-Romdhame F., Obeid S. (2024). Mediating effect of psychological distress and mindful eating behaviors between orthorexia nervosa and academic self-efficacy among Lebanese university female students. BMC Public Health.

[30] Dobos B., Berki T., Mellor D., Piko B.F. (2024). Mindful eating and orthorexia nervosa: How do they interact?. Nutr. Bull..

[31] Cerolini S., Vacca M., Zagaria A., Donini L.M., Barbaranelli C., Lombardo C. (2022). Italian adaptation of the Dusseldorf Orthorexia Scale (I-DOS): Psychometric properties and prevalence of orthorexia nervosa among an Italian sample. Eat. Weight. Disord..

[32] Falgares G., Costanzo G., Manna G., Marchetti D., Barrada J.R., Roncero M., Verrocchio M.C., Ingoglia S. (2023). Healthy orthorexia vs. orthorexia nervosa: Italian validation of the Teruel Orthorexia Scale (TOS). Eat. Weight. Disord..

[33] Anversa R.G., Muthmainah M., Sketriene D., Gogos A., Sumithran P., Brown R.M. (2021). A review of sex differences in the mechanisms and drivers of overeating. Front. Neuroendocrinol..

[34] Augimeri G., Marchese M., Plastina P., Bonofiglio D. (2025). Examining Associations Among Orthorexia Nervosa and Anthropometric Factors and Lifestyle Habits in an Italian University Community. Nutrients.

[35] Lotfi K., Saneei P., Hajhashemy Z., Esmaillzadeh A. (2022). Adherence to the Mediterranean Diet, Five-Year Weight Change, and Risk of Overweight and Obesity: A Systematic Review and Dose-Response Meta-Analysis of Prospective Cohort Studies. Adv. Nutr..

[36] Agnoli C., Sieri S., Ricceri F., Giraudo M.T., Masala G., Assedi M., Panico S., Mattiello A., Tumino R., Giurdanella M.C. (2018). Adherence to a Mediterranean diet and long-term changes in weight and waist circumference in the EPIC-Italy cohort. Nutr. Diabetes.

[37] Mendez M.A., Popkin B.M., Jakszyn P., Berenguer A., Tormo M.J., Sanchez M.J., Quiros J.R., Pera G., Navarro C., Martinez C. (2006). Adherence to a Mediterranean diet is associated with reduced 3-year incidence of obesity. J. Nutr..

[38] Schröder H., Marrugat J., Vila J., Covas M.I., Elosua R. (2004). Adherence to the traditional Mediterranean diet is inversely associated with body mass index and obesity in a Spanish population. J. Nutr..

[39] Buckland G., Bach A., Serra-Majem L. (2008). Obesity and the Mediterranean diet: A systematic review of observational and intervention studies. Obes. Rev..

[40] Papadaki A., Nolen-Doerr E., Mantzoros C.S. (2020). The effect of the Mediterranean diet on metabolic health: A systematic review and meta-analysis of controlled trials in adults. Nutrients.

[41] Esposito K., Kastorini C.M., Panagiotakos D.B., Giugliano D. (2011). Mediterranean diet and weight loss: Meta-analysis of randomized controlled trials. Metab. Syndr. Relat. Disord..

[42] Augimeri G., Lofaro D., Vivacqua A., Barone I., Giordano C., Morelli C., Conforti D., Sisci D., Catalano S., Bonofiglio D. (2025). Associations among skin carotenoids, anthropometric parameters and healthy lifestyle behaviors in young adults: A cross-sectional, population-based study. J. Transl. Med..

[43] Augimeri G., Avolio E., Caparello G., Galluccio A., De Rose D., Vivacqua A., Morelli C., Barone I., Catalano S., Ando S. (2023). Serum from Adolescents with High Polyphenol Intake Exhibits Improved Lipid Profile and Prevents Lipid Accumulation in HepG2 Human Liver Cells. Oxid. Med. Cell Longev..

[44] Augimeri G., Soto M., Ceraudo F., Caparello G., Villegas Figueroa M., Cesario M., Caputi L.S., Calderon B., Bonofiglio D. (2024). Differences of skin carotenoids and adherence to the Mediterranean Diet pattern in adults from Southern Italy and Dominican Republic. J. Transl. Med..

[45] Augimeri G., Gelsomino L., Germano M., Tripepi G., Bonofiglio D., Bonofiglio R. (2026). Skin Carotenoid Score as a Potential Early Biomarker of Metabolic Syndrome Risk in Adolescents. Nutrients.

[46] Caparello G., Groccia G.D., Ceraudo F., Cesario M., Bonofiglio R., Augimeri G., Bonofiglio D. (2023). Association between Skin Carotenoid Score Measured with Veggie Meter((R)) and Adherence to the Mediterranean Diet among Adolescents from Southern Italy. Nutrients.

[47] Damigou E., Georgoulis M., Chrysohoou C., Barkas F., Vlachopoulou E., Adamidis P.S., Kravvariti E., Tsioufis C., Pitsavos C., Liberopoulos E. (2024). Mediterranean-Type Diet Adherence and Body Mass Index through 20 Years of Follow-Up: Results from the ATTICA Cohort Study (2002–2022). Nutrients.

